# Central obesity and normal-weight central obesity among adults attending healthcare facilities in Buffalo City Metropolitan Municipality, South Africa: a cross-sectional study

**DOI:** 10.1186/s41043-017-0133-x

**Published:** 2017-12-28

**Authors:** Eyitayo Omolara Owolabi, Daniel Ter Goon, Oladele Vincent Adeniyi

**Affiliations:** 1 Department of Nursing Science, Faculty of Health Sciences, University of Fort Hare, East London, 5271 South Africa; 2 Department of Family Medicine, Faculty of Health Sciences, Walter Sisulu University/Cecilia Makiwane Hospital, East London Hospital Complex, East London, South Africa

**Keywords:** Central obesity, Overall obesity, Waist circumference, Waist-to-hip ratio, Waist-to-height ratio, NICE BMI-WC composite index, Health risk, Abdominal obesity

## Abstract

**Background:**

Central obesity (CO) confers a significant threat on the cardio-metabolic health of individuals, independently of overall obesity. Disparities in the measures of fat distribution lead to misclassification of individuals who are at risk of cardio-metabolic diseases. This study sought to determine the prevalence and correlates of central obesity and normal-weight central obesity among adults attending selected healthcare facilities in Buffalo City Metropolitan Municipality (BCMM), South Africa, assess their health risk and examine the association between central obesity and cardio-metabolic diseases among adults with normal weight, measured by body mass index (BMI).

**Methods:**

A cross-sectional survey of 998 adults was carried out at the three largest outpatient clinics in BCMM. Overall and central obesity were assessed using BMI, waist circumference (WC), waist-to-hip ratio (WHR) and waist-to-height ratio (WHTR). The WHO STEPwise questionnaire was used for data collection. Blood pressure and blood glucose were measured. Normal-weight central obesity was defined as CO among individuals with normal weight, as assessed by BMI. Health risk levels were assessed using the National Institute for Health and Clinical Excellence (NICE) BMI-WC composite index. Bivariate and multivariate analyses were used to determine the prevalence of CO, normal-weight central obesity and the predictors of CO.

**Results:**

The mean age of participants was 42.6 (± 16.5) years. The prevalence of CO was 67.0, 58.0 and 71.0% by WC, WHR and WHTR, respectively. The prevalence of normal-weight central obesity was 26.9, 36.9 and 29.5% by WC, WHR and WHTR, respectively. About 41% of the participants had a very high health risk, 13% had increased risk or high risk and 33% had no health risk. Central obesity was significantly associated with hypertension but not associated with diabetes among those with normal weight (by BMI). Female sex, age over 30 years, marriage, secondary or tertiary level of education, non-smoking status, diabetes and hypertension significantly predicted central obesity among the study participants.

**Conclusion:**

The prevalence of central obesity among the study participants is high, irrespective of the defining criteria. One in three adults of normal weight had central obesity. Body mass index should therefore not be used alone for clinical assessment by healthcare workers in the study setting.

## Background

Obesity is a medical condition characterised by an abnormal fat accumulation which is detrimental to health [1, 2]. Excessive intake of energy-dense foods, physical inactivity and genetic susceptibility are known causative factors of obesity [3, 4]. Excess weight is one of the leading causes of morbidity and mortality and is exponentially increasing worldwide [5, 6]. Currently, over half a billion adults are considered obese [2]. Worryingly, the obesity epidemic is growing faster in developing countries [7–9], and South Africa has a higher prevalence of obesity than most other developing countries [6, 10].

Although overall obesity confers a significant threat to the health of individuals, the distribution of body fat is also of great importance in determining this threat [11–13]. Central obesity has been recognised as an independent risk factor for cardio-metabolic diseases and a better predictor of cardiovascular risk than overall obesity [14, 15]. Central obesity is a component of metabolic syndrome and plays a vital role in the pathogenesis of cardiovascular diseases (CVD) and certain cancers by stimulating mediating factors such as insulin resistance, dyslipidaemia and systematic inflammation, even among individuals with normal weight [16]. This risk increases with an increase in abdominal fat [17].

Although BMI is the most common measure of overall obesity [18], it would be misleading to use it as a health risk indicator alone, because BMI alone results in misclassification and underestimation of the at-risk population; individuals with a normal weight are sometimes centrally obese [19, 20]. Such individuals are generally not counselled to take action about their health. Ideally, a direct measure of abdominal fat involves the use of imaging techniques such as the computed tomography scan and magnetic resonance imaging [21]. However, these gold-standard techniques are expensive, time-consuming and impractical in resource-limited settings such as South Africa and in large epidemiological surveys [8]. In this wise, proxy measures of anthropometric indicators such as waist circumference (WC), waist-to-hip ratio (WHR) and waist-to-height ratio (WHTR) are used to assess central fat distribution. Studies have shown that these measures are strongly associated with all-cause CVD and cancer mortality, independently of BMI [22–25]. Also, the addition of anthropometric indicators of central obesity to BMI in all clinical assessments has been recommended by International Health Organizations, such as the National Institute for Health and Clinical Excellence and the National Heart, Lung and Blood Institute [26, 27]. Some authors also recommend an assessment of central obesity even among individuals with normal weight [28, 29] in order to improve assessment of cardio-metabolic risk.

Anecdotal evidence, however, shows that such practices are not in place in primary healthcare facilities in South Africa where preventative health measures are implemented. Although the Department of Health stated that a measure of central obesity be used alongside the BMI [30], anecdotal evidence however shows that central obesity is generally only assessed among overweight and obese patients, and ignored when it comes to normal-weight individuals, whose pattern of fat distribution is important in the clinical and public health context. Central obesity among adults has been scantily investigated in South Africa [31–33], and there is a dearth of information on the prevalence of central obesity among normal-weight individuals in South Africa. To the best knowledge of the authors, no study has assessed the prevalence of normal-weight central obesity and its association with cardio-metabolic diseases among adults in South Africa. Such information will be useful to evaluate potential health risks and plan for interventions. Viewed in this context, the present study has a threefold objective: (1) to determine the prevalence and correlates of central obesity and normal-weight central obesity among adults attending selected healthcare facilities in BCMM, South Africa using various proxy measures, (2) to examine their health risk and (3) to determine the association between central obesity and cardio-metabolic diseases among adults with normal weight, measured by BMI.

## Methods

### Study area and design

This study analysed data from the Buffalo City Metropolitan Municipality (BCMM) Non-Communicable Diseases Surveillance Study. Details regarding the methodology of the study have been previously explained [34]. Briefly, the three largest outpatient clinics serving the residents of BCMM, South Africa, were selected. These clinics provide primary healthcare services for the 755 200 residents of Buffalo City Municipality in the Eastern Cape Province [35].

A sample size of 1107 participants was estimated across the three study sites (369 per site), based on the estimated non-communicable disease prevalence rate of 40% in South Africa, with a sampling error of 5% and a 95% confidence level.

Outpatients and relatives were informed about the study objectives and the procedure as well as their right to voluntary participation and confidentiality. Afterwards, all ambulatory individuals, majorly patients and very few family members (20) who showed interest, fulfilled the inclusion criteria and attended the study settings during the period of study were recruited into the study. This study was conducted in April and May, 2016. A convenience sampling method was utilised.

Eligibility criteria included age ≥ 18 years, attendance at the outpatient clinics, and 8 h of fasting prior to recruitment into the study. Patients who were psychotic, debilitated, pregnant or handicapped in any form to the point that obtaining anthropometric measurement would be difficult were excluded from the study. A consecutive sample of 1107 participants participated in the study. However, 109 participants were excluded as a result of incomplete data. Thus, 998 participants were included in the analysis.

### Study instrument

Participants were interviewed using the previously validated WHO STEPwise questionnaire [36] which comprises three major items: demographic data, behavioural data and measurements. All interviews were conducted by trained research assistants.

### Ethical considerations

Ethical approval was obtained in accordance with the Helsinki II Declaration from the University of Fort Hare Research Ethics Committee and the Eastern Cape Department of Health (Reference number; GOO061SOLO01). The management of the sub-district Department of Health as well as the heads of the respective health facilities gave permission prior to data collection. All participants provided written informed consent to participate in this study. Anonymity and confidentiality were ensured.

### Data collection procedure

Data were obtained by personal interviews for demographic and behavioural characteristics and measurements of blood pressure, blood glucose and anthropometric parameters. Demographic variables included items on sex, age, marital status, level of education, employment status and average monthly income. The socioeconomic factors were measured by assessing the average monthly income, level of education and employment status. Participants were categorised as low-income earners if they earned 140USD or less per month, middle-income earners if they earned 140-350USD and high-income earners if they earned above 350USD. Level of education was obtained by self-reporting of the highest grade level attained in school, with participants categorised as having no formal education, primary (Grades 1–7), secondary (Grades 8–12) or tertiary (post-secondary) education. Participants were defined as unemployed if they reported that they were not employed in either the formal or informal sectors.

The following behavioural variables were obtained by self-reporting: smoking, alcohol use and physical activity. Smoking and alcohol use were assessed by self-reporting on the use of any tobacco product or alcoholic drink in the past 30 days. Participants’ levels of physical activity were obtained by self-reported engagement in moderate (yes/no) or vigorous (yes/no) intensity exercise.

### Measurements

Blood pressure (systolic and diastolic) was measured in accordance with standard protocols [37] with a validated Microlife BP A100 Plus model. Hypertension was defined as an average of two systolic blood pressure measurements of ≥ 140 mmHg or a diastolic blood pressure of ≥ 90 mmHg, in accordance with the Seventh Joint National Committee (JNC 7) guidelines or a history of hypertension or use of hypertension medication(s). The fasting blood glucose of each participant was measured with a validated ACCU-CHEK glucose monitoring apparatus in fasting state. Participants were diagnosed as having diabetes if their fasting blood glucose level was equal to or greater than 7.0 mmol/L, or if they were on current medication for diabetes [38].

Anthropometric measurements followed the standard anthropometric methods of the International Society for the Advancement of Kinanthropometry (ISAK) [39]. Body weight was measured in light clothes to the nearest 0.01 kg in the standing position using a Soehnle scale (Soehnle-Waagen Gmbh Co., Muurhardt, Germany), and height was measured to the nearest 0.1 m by stadiometer in standing position with closed feet, without shoes. Waist circumference was measured at the level of the narrowest point between the lower costal border and the iliac crest at the end of a normal exhalation with the arms relaxed at the sides. The hip circumference was measured at the widest circumference of the buttock with a non-extensible tape to the nearest 0.1 cm. All measurements were taken by a trained research assistant to ensure uniformity.

### Definition of overall and central obesity

Body mass index (BMI) was calculated as weight in kilogrammes divided by height in square metres (kg/m^−2^). BMI was categorised in accordance with WHO [18] as underweight (< 18.5 kg/m^−2^), normal (18.5–24.9 kg/m^−2^), overweight (25.0–29.9 kg/m^−2^) and obese (> 30.0 kg/m^−2^). Central obesity was defined according to the WHO criteria [18]: WC ≥ 94 cm for men and ≥ 80 cm for women or waist-to-hip ratio (WHR) ≥ 0.90 in men and ≥ 0.85 in women and a WHTR of > 0.50 [20]. Normal-weight central obesity was defined as central obesity (CO) in participants with normal weight (by BMI).

### Assessment of health risk

The assessment of health risk was based on a BMI-WC matrix developed by the National Institute of Health and Clinical Excellence for managing overweight and obesity [26, 28]. According to this matrix, a person is classified as underweight for a BMI < 18.5 kg m^−2^, healthy weight for a BMI of 18.5–24.9 kg m^−2^, overweight for a BMI of 25–29.9 kg m^−2^, obese for a BMI of 30–39.9 kg m^−2^ and very obese for a BMI > 40 kg m^−2^. Waist circumference was classified as:

- Low: WC < 94 cm in men and < 80 cm in women
- High: WC of 94–102 cm in men and 80–88 cm in women
- Very high: WC > 102 cm in men and > 88 cm in women

The NICE BMI-WC matrix categorised health risks as follows:

- No increased risk, defined as healthy weight combined with low or high WC and overweight with low WC
- Increased health risk, defined as healthy weight with very high WC, overweight with high WC, and obese with low WC
- High risk, defined as overweight with a very high WC and obese with high WC
- Very high risk, defined as obese with very high WC and very obese with any category of WC (Table 1)

**Table 1:**
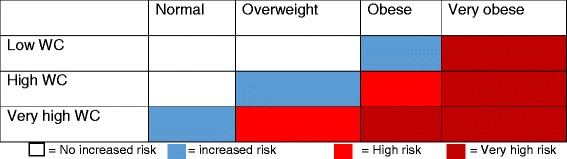
Health risk groups using BMI-WC index

### Statistical analysis

Data were expressed as mean values ± standard deviations (SD) for continuous variables. Counts (frequencies = *n*) and proportions (%) were reported for categorical variables. A bivariate analysis was used to examine variables that have a significant association with central obesity. The significant variables were included in the binary logistic regression and were adjusted for confounding factors. Analysis was carried out at a 95% confidence level. A *p* value of < 0.05 was considered statistically significant. Statistical analysis was performed with the Statistical Package for Social Science (SPSS), version 21 for Windows (SPSS Inc., Chicago, IL, USA).

## Results

The mean age of participants was 42.6 (SD ± 16.5) years. The majority of the participants were black (98.1%), female (67.8%) and single (63.9%) and had at least secondary level of education (69.7%). About half of the participants had no income (44.6%) and were unemployed (47.7%), while only a few (7.5%) participants earned above 400USD monthly.

### Prevalence of central obesity and normal-weight central obesity

**Fig. 1:**
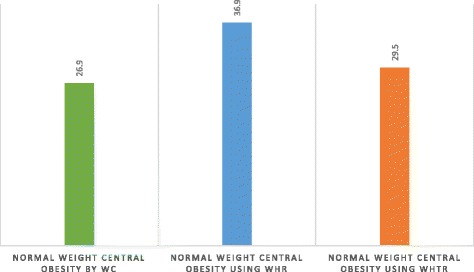
Prevalence of normal weight central obesity using different anthropometric measure

The prevalence of underweight, normal, overweight and obesity were 4.0, 27.0, 24.0 and 46.0%, respectively. The prevalence rates of central obesity by WC, WHR and WHTR were 66.6, 57.8 and 71.4%, respectively. Of the 271 participants classified as normal weight using BMI, the prevalence of central obesity was 26.9, 36.9 and 29.5% by WC, WHR and WHtR, respectively (Fig. 1).

### BMI distribution across WC levels of risk

As shown in Table 2, 26.9% of participants with a healthy weight, using BMI, had either a high-risk or very high-risk waist circumference. Thirty percent of the overweight participants had a low-risk WC while the majority of the obese and very obese participants also had a very high-risk waist circumference.

**Table 2:** Health risks of the study participants

BMI categories	Low risk WC^π^	High risk WC*	Very high risk WC^≠^
Underweight	35 (97.2)	1 (2.8)	0 (0.0)
Healthy weight	198 (73.1)	48 (17.7)	25 (9.2)
Overweight	72 (30.1)	77 (32.2)	90 (37.7)
Obese	24 (6.9)	36 (20.9)	286 (82.7)
Very obese	4 (3.8)	10 (9.6)	90 (86.5)

*WC* waist circumference, *WHR* waist-to-hip ratio, *WHTR* waist-to-height ratio

^π^WC < 94 cm for men and < 80 cm for women

*WC 94–102 cm for men and 80–88 cm for women

^≠^WC > 102 cm for men and > 88 cm for women

### Determinants of central obesity

Sex, age, educational level, marital category, income, smoking, alcohol use, diabetes and hypertension were significantly associated with central obesity using the WC (Table 3). However, after adjusting for confounding factors (income, alcohol use and employment status), only female sex, age over 30 years, marriage, secondary or tertiary level of education, not smoking, diabetes and hypertension were significant predictors of abdominal obesity among the study participants. Female participants were 11 times more likely to be abdominally obese than their male counterparts. Participants who do not smoke, have a secondary or tertiary level of education and are above 30 years of age, married, hypertensive and diabetic were twice as likely to have abdominal obesity than those who smoked, had no or primary level of education, were under 30 years, not hypertensive and not diabetic (Table 4).

**Table 3:** Bivariate analysis showing factors associated with central obesity

Variables	CO (WC)	Not CO	*p* value	CO (WHR)	Not CO	*p* value	CO (WHTR)	Not CO	*p* value
Sex									
Male	114 (35.5)	207 (64.5)	< 0.001	166 (51.7)	155 (48.3)	0.005	172 (53.6)	149 (46.4)	< 0.001
Female	549 (81.3)	126 (18.7)		409 (60.7)	265 (38.3)		539 (79.9)	136 (20.1)	
Educational level									
No formal schooling	79 (54.1)	67 (45.9)	0.007	87 (59.6)	59 (40.4)	0.251	93 (63.7)	53 (36.3)	0.144
Grades 1 to 7	104 (67.1)	51 (32.9)		100 (64.5)	55 (35.5)		115 (74.2)	40 (25.8)	
Grades 8 to 12	399 (68.8)	181 (31.2)		323 (55.8)	256 (44.2)		422 (72.8)	158 (27.2)	
Tertiary	81 (70.4)	34 (29.6)		65 (56.5)	50 (43.5)		81 (70.4)	34 (29.6)	
Smoking									
Yes	51 (34.0)	99 (66.0)	< 0.001	71 (47.3)	79 (52.7)	0.003	66 (56.0)	84 (56.0)	< 0.001
No	612 (72.3)	234 (27.7)		504 (59.6)	341 (40.4)		645 (76.2)	201 (23.8)	
Alcohol use									
Yes	171 (53.8)	147 (46.2)	< 0.001	159 (50.0)	159 (50.0)	< 0.001	195 (61.3)	123 (38.7)	< 0.001
No	486 (72.8)	182 (27.2)		411 (61.6)	256 (38.4)		509 (76.2)	159 (23.8)	
Hypertension									
Yes	363 (74.1)	127 (25.9)	< 0.001	332 (67.9)	157 (32.1)	< 0.001	396 (80.8)	94 (19.2)	< 0.001
No	300 (59.3)	206 (40.7)		243 (48.0)	263 (52.0)		315 (62.3)	191 (37.7)	
Diabetes									
Yes	193 (79.4)	50 (20.6)	< 0.001	179 (74.0)	63 (26.0)	< 0.001	209 (86.0)	34 (14.0)	< 0.001
No	470 (62.4)	283 (37.6)		396 (52.6)	357 (47.4)		502 (66.7)	251 (33.3)	
Physical activity									
Yes	305 (68.2)	142 (31.8)	0.174	184 (41.3)	236 (43.0)	0.314	318 (71.1)	129 (28.9)	0.466
No	358 (65.2)	191 (34.8)		262 (58.7)	313 (57.0)		393 (71.6)	156 (28.4)	
Marital status									
Never married	386 (60.8)	249 (39.2)	< 0.001	312 (49.2)	322 (50.8)	< 0.001	411 (64.7)	224 (35.3)	< 0.001
Ever married	250 (76.2)	78 (23.8)		239 (72.9)	89 (27.1)		271 (82.6)	57 (17.4)	
Income categories									
≤ R2000	94 (61.8)	58 (38.2)	0.011	90 (59.2)	62 (40.8)	0.200	106 (69.7)	46 (30.3)	0.060
2001–4999	268 (75.1)	89 (24.9)		233 (65.3)	124 (34.7)		282 (79.0)	75 (21.0)	
5000 and above	53 (70.7)	22 (29.3)		42 (56.0)	33 (44.0)		60 (80.0)	15 (20.0)	
Age									
< 35 years	222 (57.7)	164(42.3)	< 0.00	159 (41.4)	225 (58.6)	< 0.001	233 (69.5)	152 (39.5)	< 0.001
35 and above	441 (72.2)	170(27.8)		416 (68.1)	195 (31.9)		478 (78.2)	133 (21.8)	
Employment status									
Government employee	46 (73.0)	17 (27.0)	< 0.001	35 (55.6)	28 (44.4)	< 0.001	52 (82.5)	11 (17.5)	< 0.001
Non-government employee	142 (61.5)	89 (38.5)		136 (58.9)	95 (41.1)		158 (68.4)	73 (31.6)	
Self-employed	37 (59.7)	25 (40.3)		35 (56.5)	27 (43.5)		39 (61.9)	23 (37.1)	
Student	48 (48.5)	51 (51.5)		27 (27.3)	72 (72.7)		48 (48.5)	51 (51.5)	
Unemployed	340 (71.7)	134 (28.3)		294 (62.2)	179 (37.8)		360 (75.9)	114 (24.1)	
Retired	49 (74.2)	17 (25.8)		47 (71.2)	19 (28.8)		53 (80.3)	13 (19.7)	
Comorbidity (hypertension, diabetes)									
Yes	217 (99.5)	1 (0.5)	< 0.001	178 (82.0)	39 (18.0)	< 0.001	216 (99.1)	2 (0.9)	< 0.001
No	332 (42.7)	446 (57.3)		397 (51.0_	381 (49.0)		495 (63.6)	283 (36.4)	

*CO* central obesity, *WC* waist circumference, *WHR* waist-to-hip ratio, *WHTR* waist-to-height ratio

**Table 4:** Logistic regression showing predictors of central obesity

Variables	Beta	S.E	Wald	OR (95% CI)	*p* value
Sex					
Female	2.4	0.2	152.6	11.1 (7.6–16.2)	< 0.001
Male (reference)					
Smoking					
No	0.8	0.2	10.8	2.1 (1.4–3.4)	0.001
Yes (reference)					
Level of education					
Secondary or tertiary level of education	0.7	0.2	13.4	2.0 (1.4–2.9)	< 0.001
No or primary education (reference)					
Age					
Above 30 years	0.8	0.2	13.7	2.1(1.4–3.2)	< 0.001
30 years or below (reference)					
Marital status					
Married	0.7	0.2	10.2	1.9 (1.3–2.9)	0.001
Never married (reference)					
Hypertension					
Yes	0.7	0.2	15.6	2.1 (1.5–3.0)	< 0.001
No (reference)					
Diabetes					
Yes	0.8	0.2	12.1	2.2 (1.4–3.5)	< 0.001
No (reference)					

### Health risk assessment

Using the NICE BMI-WC composite index, almost half (41%) of the study participants had a very high health risk, 13% had either an increased risk or a high risk while 33% had no increased health risk.

### Association between normal-weight central obesity and cardio-metabolic diseases

Central obesity was significantly associated with hypertension but not diabetes mellitus (DM) among participants with normal weight, measured by BMI. The prevalence of hypertension among those with normal-weight central obesity was 46 and 43.8% compared to 25.7 and 28.8% among those with normal weight with no central obesity using WHR and WHTR, respectively. Irrespective of the defining criteria, those with normal-weight central obesity had a higher prevalence of DM than those with normal weight without central obesity, although the difference is not statistically significant (Table 5).

**Table 5:** Association of normal weight by BMI in predicting hypertension and DM versus central obesity proxy measures

Variables	Hypertensive (%)	*p* value	Diabetic (%)	*p* value
WC				
Normal weight, no central obesity	65(32.8)	0.467	26 (13.1)	0.522
Normal-weight central obesity	25(34.8)		10 (13.7)	
WHR				
Normal-weight, no central obesity	44(25.7)	0.001	19 (11.1)	0.117
Normal-weight central obesity	46(46.0%)		17 (17.0)	
WHTR				
Normal weight, no central obesity	55(28.8)	0.013	24 (12.6)	0.360
Normal-weight central obesity	35(43.8)		12 (15.0)	

Hypertensive = blood pressure ≥ 140/90 mmHg or history of hypertension or hypertension treatment; diabetes = fasting blood glucose ≥ 7.0 mmol/L or a history of diabetes or diabetes treatment

*WC* waist circumference, *WHR* waist-to-hip ratio, *WHTR* waist-to-height ratio

## Discussion

Comparing the prevalence of CO is a challenging task because of differences in the definition used by various authors. Irrespective of the defining criteria, the prevalence of CO found in this study population is high. The prevalence of CO by WC, which is the most commonly used criterion, is higher than the reported prevalence among adults in other countries: Nigeria [21, 40]; Iran [41]; USA [42], Portugal [43]; Spain [44] and China [45]. The prevalence reported by these studies ranged from 33.8% among black, outpatient Nigerian adults to 58.4% among Spanish adults. Although, the participants in these studies have different demographic characteristics such as race and income level, mostly higher than the current study, and place of residence, majorly urban, even so, the prevalence reported in this study is high. The prevalence found in this study is comparable only to the reported prevalence of 68.2% among South African nurses [31] with a higher income level and South African blacks (60%) living in an urban area in Cape Town [32].

A national survey conducted in 2002 among South African adults reported a lower prevalence of CO (51.4%) than the reported prevalence in this study (66.6%) [33]. This finding reinforces the postulation that South Africa is at the forefront of the obesity epidemic. Although genetics play an important role in the obesity epidemic [3], other factors play an important role too, including nutritional transition as a result of urbanisation and westernisation, which promotes the neglect of traditional healthy diets, less physical activity and the consumption of westernised, energy-dense foods and sugar-sweetened beverages [46, 47]. Also, a poor perception regarding weight still persists among African adults; sometimes, a large body size is erroneously perceived as a sign of affluence [21, 32, 48]. Considering the deleterious effect of central obesity, there is an urgent need to implement measures that can help to curtail this rising health concern, by creating awareness on the health implications of excessive visceral fat (central obesity) among the populace.

The prevalence of normal-weight central obesity found in this study ranged from 26.9 to 36.9%, depending on the criteria used. This finding is higher than the reported prevalence of normal-weight central obesity among Thai health workers, at 15.4% [29], and Chinese adults, at 13.9% [49]. The higher prevalence of normal-weight central obesity found in this study is suggestive of the need to include anthropometric indices in the measurement of excessive body weight, other than BMI alone, as BMI alone might result in misclassifications and an underestimation of at-risk individuals. This being the case, such individuals would not normally be offered the appropriate health education and prompt intervention to manage and/or prevent the development of cardio-metabolic complications. This therefore points to the need for district health managers to revise the clinical practice guidelines for managing chronic illnesses, by including at least one anthropometric measurement of abdominal obesity as one of the vital signs to monitor at health facilities, particularly at the primary healthcare centres where preventative measures are practised. This challenge has already been identified in developed countries such as the USA and the UK, where the assessment of abdominal obesity in addition to BMI has been added to their clinical guidelines [26]. This is the right time for South Africa to reinforce the need for this form of assessment, as BMI alone is no longer sufficient.

Almost half (41%) of the study participants were classified as being in the very high health risk group, 13% were classified as having either increased health risk or high health risk and only 33% had no increased health risk, using the NICE BMI-WC composite index [26, 28]. This is crucial information which could easily be obtained at health facilities if anthropometric measurements such as the WC were taken alongside BMI. This would afford clinicians the opportunity to offer care and attention to their patients based on their health risk classification. Also, the majority of the at-risk population could be promptly identified and given appropriate care, thus reducing the chances of misclassification of such individuals. Such strategies could help reduce the burden of central obesity and prioritise cardio-metabolic health screening, even in the presence of limited resources.

Only female sex, age over 30 years, being married, high level of education, not smoking, diabetes and hypertension were independent predictors of abdominal obesity. The association between age, marriage, level of education, female sex and abdominal obesity has been documented by several studies [31, 42, 50]. The higher likelihood of being obese among females can be linked to women’s lower engagement in physical activity as well as the physiological changes which occur during their reproductive years [51–53]. Nonetheless, the high prevalence of obesity found among females might not entirely depend on these factors, as engagement in physical activity was not even associated with CO in this study; genetics, too, could play a vital role in the development of CO [3, 54]. This could also be a plausible reason for the higher prevalence of obesity among older and married participants. In addition to this, Janghorbani et al. [50] highlighted changes in dietary patterns as well as a lower emphasis on being attractive after marriage as some of the reasons for the observed higher prevalence of CO among married individuals. Strategic plans tailored towards those at higher risk of developing central obesity are urgently needed in this setting.

An inverse association was found between smoking and abdominal obesity. This is consistent with previous studies [50, 55]. Gallus et al. [54] and Mackay, Gray and Pell [55] reported inadvertent weight loss among smokers; Janghorbani et al. [50] ascribed it to smoking’s effect on metabolic rate, energy intake and storage and energy expenditure. Even so, the cumulative effect of smoking on health is detrimental, irrespective of its effect on weight loss.

Also, this study found a higher prevalence of CO among participants with a secondary or tertiary level of education. This finding needs to be interpreted with caution. The majority of the study participants had a low or average level of education. Central obesity has been shown to be associated with low education and socioeconomic status, which are characterised by poor health behaviours [32, 43, 56].

The present study found an association between central obesity and other cardio-metabolic diseases such as diabetes and hypertension, and clustering of both hypertension and diabetes. This supports the findings of several studies [57, 58], which identified central obesity as a risk factor for hypertension, diabetes type 2 and co-morbidities, even in the absence of overall obesity. Central obesity stimulates the development of mediating factors such as insulin resistance, glucose intolerance, endothelial dysfunction and systematic inflammation, which contribute to the development of chronic diseases. Among those with normal weight by BMI, central obesity was also associated with hypertension but not with diabetes, although those with central obesity still had a higher prevalence of diabetes. Central obesity among normal-weight individuals has been shown to be associated with greater cardiovascular risk and mortality than is found among normal weight individuals without central obesity [59]. This highlights the need to include measures of central obesity in all clinical assessments in this study setting. Measures aimed at reducing obesity should be prioritised in order to curb the growing prevalence of chronic diseases, which impacts greatly on the already over-burdened healthcare system. The time to act is now or never.

### Strengths and limitations

To the best knowledge of the authors, this is the first study reporting on the prevalence of central obesity and normal-weight central obesity in the Eastern Cape Province of South Africa. Also, this is the first study assessing the health risks related to central obesity and the association between central obesity and cardio-metabolic diseases among those with normal weight, by BMI, in South Africa. Such important information is essential for crafting effective public health policies and clinical guidelines. The large sample size, the use of standard procedures and the use of various anthropometric measures to assess central obesity further give credence to the findings of this study. However, the cross-sectional nature of this study, convenience sampling and the use of self-reported lifestyle behaviours are obvious limitations. The inability to carry out a confirmatory test for the diagnosis of diabetes and repeated measures of blood pressure for diagnosing hypertension are additional limitations. The authors did not examine the association between normal-weight central obesity and dyslipidaemia, another cardio-metabolic disease. Also, the findings of this study might not be representative of the entire Eastern Cape or South African adults; thus, it cannot be generalised to those population. However, this study gives a snapshot of the burden of central obesity in individuals with normal weight by BMI and a health risk assessment of adults in this setting.

## Conclusion

There is a high prevalence of central obesity among adults in this setting. Also, one in every three adults considered to have a normal weight using the BMI alone is centrally obese. Almost half of the study participants had a very high health risk, and central obesity was associated with hypertension, even among those with normal weight by BMI. Female sex, increasing age, average level of education, marriage, smoking, diabetes and obesity were significant predictors of central obesity among participants in this study. This study established that the use of BMI alone as a measure of obesity is insufficient for assessing health risk. There is an urgent need to include any anthropometric measure of central obesity in the vital signs measured in clinical practice.

## Abbreviations

BCMM: Buffalo City Metropolitan Municipality
BMI: Body mass index
BP: Blood pressure
CO: Central obesity
CVD: Cardiovascular diseases
DM: Diabetes mellitus
JNC: Joint National Committee
WC: Waist circumference
WHO: World Health Organization
WHR: Waist-to-hip ratio
WHtR: Waist-to-height ratio
